# Reliability and agreement of the IsoKai isokinetic lift test – A test used for admission to the Swedish Armed Forces

**DOI:** 10.1371/journal.pone.0209419

**Published:** 2018-12-19

**Authors:** Tony Bohman, Matthias Tegern, Alexandra Halvarsson, Lisbet Broman, Helena Larsson

**Affiliations:** 1 Department of Neurobiology, Care Sciences and Society, Division of Physiotherapy, Karolinska Institutet, Stockholm, Sweden; 2 Department of Community Medicine and Rehabilitation, Umeå University, Umeå, Sweden; 3 Allied Health Professionals Function, Karolinska University Hospital, Stockholm, Sweden; 4 Swedish Armed Forces, Headquarters, Medical Services, Stockholm, Sweden; University of L'Aquila, ITALY

## Abstract

This study was performed to evaluate the reliability and agreement of the IsoKai isokinetic lift test as it is currently administered in admission to the Swedish Armed Forces. The study included an intrarater (n = 534) and interrater reliability sample (n = 137), of Swedish male conscripts who performed the test on two test occasions about two hours apart. Two-to-four lifts were performed at each occasion, and the highest mean (IsoKai_MF_) and peak force (IsoKai_PF_) produced (N) were used for evaluation. All intraclass coefficients showed excellent reliability. The interrater analyses resulted in intraclass coefficients of 0.942 (95% CI; 0.920–0.959) and 0.858 (95% CI; 0.806–0.896) for the IsoKai_MF_ and IsoKai_PF_, respectively, while the corresponding coefficients for the intrarater analyses were 0.935 (95% CI; 0.923–0.946) and 0.865 (95% CI; 0.842–0.886). Agreement, the capability of a test to detect changes, was assessed by the standard error of measurement (SEM/SEM%) and the smallest real difference (SRD/SRD%). These estimate indicated that it is possible to achieve measurements relevant to use in real practice with the IsoKai isokinetic lift test. Bland and Altman analyses revealed no systematic errors in either sample. Based on these findings, the IsoKai isokinetic lift test is suggested to be a highly reliable test for maximal dynamic muscular strength. The test could be of use in selection procedures in order to accurately evaluate maximal dynamic muscular strength, and for evaluating longitudinal changes in strength.

## Introduction

Measuring muscular strength is often of interest and importance in many areas such as sports, rehabilitation, military settings and research [1–3]. Measures of muscular strength could either be isometric or dynamic [2]. Standard methods of measuring maximal dynamic strength include the isoinertial one-repetition maximum test (1-RM test), using external weight loading, and isokinetic strength testing performed with various commercial devices [2, 4]. In an isokinetic test, the movement velocity is held constant while the resistance adapts to the muscle force produced in every part of the movement. This is in contrast to an isoinertial strength test where the resistance is constant throughout the full movement [4, 5]. An isokinetic test is considered to be a safer alternative to an isoinertial test since the risk of overload is lower due to the adaptation of the resistance to the muscle force [2, 5]. Other benefits of isokinetic tests are the fast and easy administration of tests and their often good reliability [2, 5]. The purpose of a strength test could be to define individual physical fitness level, distinguish individuals with regard to strength capacity, or to evaluate change in strength over time in individuals or in groups [1–3]. This could, however, only be accomplished if the measures of strength are valid and reliable. The validity of a test concerns the degree to which it measures what it is intended to measure [6]. To be reliable, tests need to have a high degree of reliability and agreement, properties that set the standard for validity, and provide quantifications of the test measurement [6, 7]. Moreover, the term reliability refers to the ability of a test to differentiate between individuals, while agreement is a measure of the capability to detect changes [6, 7]. Reliability and agreement of a measure could be assessed using an inter- as well as intrarater reliability study design [6, 7]. Interrater reliability evaluates the consistency of test results at two test occasions administered by two different raters, while intrarater reliability evaluates the consistency between two test occasions administered by the same rater [6, 7].In some physically demanding occupations, muscular strength tests are used to evaluate work capacity in connection to admission for certain programs and for job selection during employment, e.g. firefighters, policemen and soldiers [8–10]. It is important that these strength tests are related to the work tasks commonly performed in the specific occupations in order to minimize the risk for disability, time off-duty and earlier retirement [8, 11]. Preferably, such tests should be safe, fast and easy to administer.

**Fig 1:**
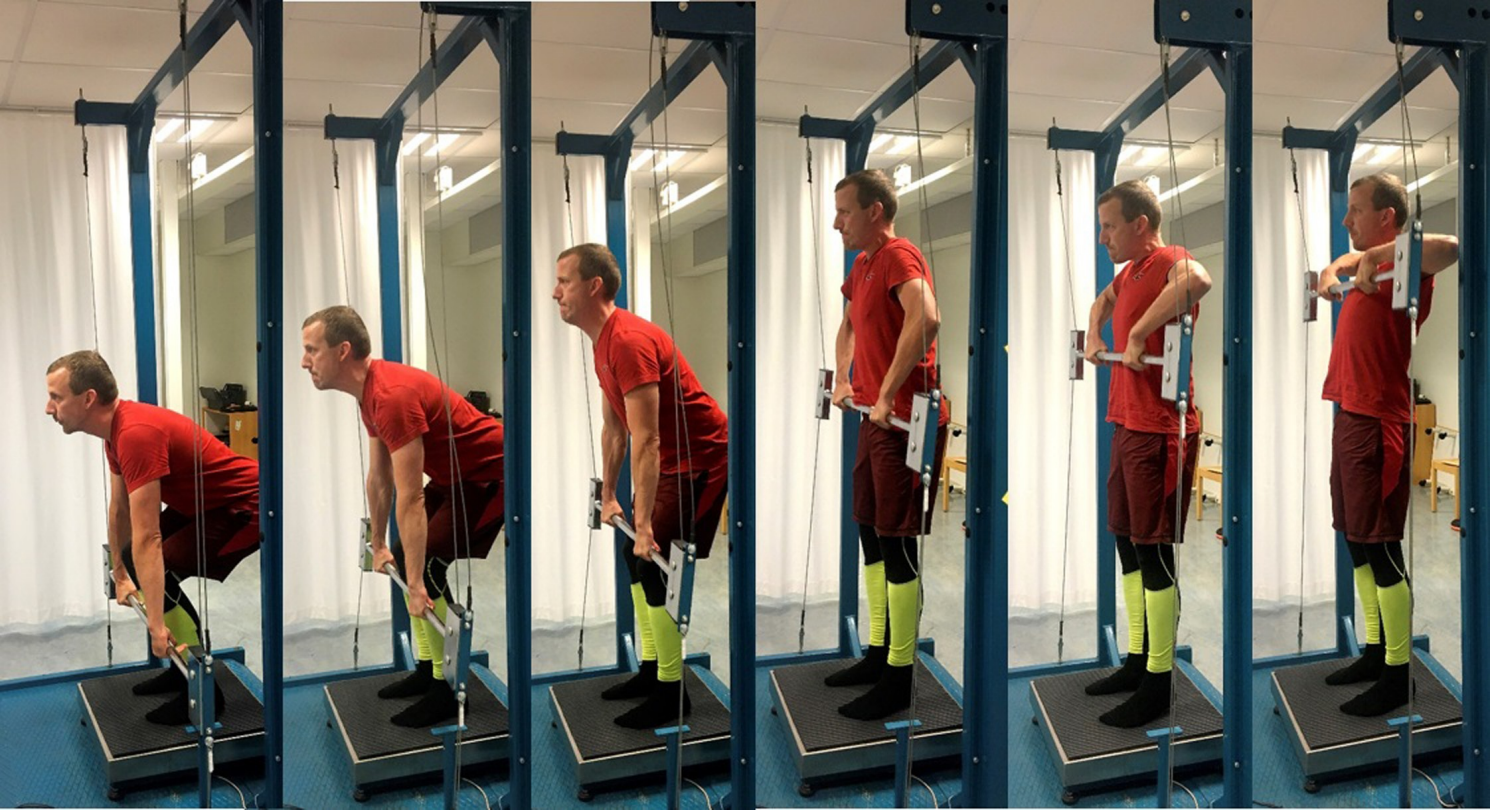
The IsoKai isokinetic lift test.

One example of such a test is the IsoKai isokinetic lift test that has, since 1995 been used to assess muscular strength on admission to service in the Swedish Armed Forces (SwAF). The IsoKai lift test was introduced as a safe and better test to predict dynamic strength needed in military work tasks than the former used isometric strength test [12, 13]. The IsoKai isokinetic lift test have excellent content validity for lifting, carrying with hands and digging, which are work tasks commonly performed during military service [14]. The test is performed in a device measuring the isokinetic mean force (IsoKai_MF_) and peak force (IsoKai_PF_), with values in Newton (N), during a maximal two-handed lift of a weight-lifting bar from “floor” to shoulder level (Fig 1).

The IsoKai_MF_ measure represents the average force produced during one lift, while the IsoKai_PF_ measure represents the maximum force produced during a lift. The IsoKai_PF_ is produced and registered when the bar reaches about hip level. Since 2013, an admission test for the SwAF and the Swedish Police includes two to four IsoKai lift attempts. The highest IsoKai_MF_ and IsoKai_PF_ registered from the lifts are used as test measures [15]. A study from 2009 showed the IsoKai test to have excellent interrater reliability when using the mean value of the IsoKai_MF_ from two lifts as outcome in a sample of Swedish conscripts [16]. However, the IsoKai test has not been assessed for reliability when using the highest IsoKai_MF_ and IsoKai_PF_ as test measures, i.e. the way it is currently used in the SwAF and the Swedish Police [15]. At this backdrop, the aim of this study was to examine the intra- and interrater reliability and agreement of the IsoKai isokinetic lift test in Swedish male conscripts using the highest registered IsoKai_MF_ and IsoKai_PF_ as measures.

## Material and methods

### Study design and study sample

The study was designed to include both an intra- and interrater reliability evaluation using two different samples. The data used to assess the intrarater reliability part of the study were collected from IsoKai isokinetic tests on male conscripts at the time of entering military service in 2002. Out of 578 eligible for test, 44 were absent due to sick-leave or other assignments, resulting in a sample of 534 conscripts. Data for the interrater reliability part were collected from another sample of 137 male conscripts, randomly selected from 601 eligible conscript at the end of their 10 months military service in 2001. At the time of data collection, the Swedish military system was based on compulsory military service for males. To be included in the study, conscripts had to be healthy and without any pain which could influence the test procedure. The IsoKai lift tests were administered by personnel from the Swedish Defence Recruitment Agency, which were well-educated in test procedures, and all had several years of experience in physical testing. Participants signed a written informed consent after receiving written information. The individual in this manuscript (Fig 1) has given written informed consent, as outlined in PLOS consent form, to publish these case details. The study was approved by the regional ethic committee in Stockholm, Gothenburg and Orebro, Sweden, Dnr 500:16 307/01. Table 1 presents the characteristics of the two study samples.

**Table 1:** Characteristics of the intrarater and interrater study samples.

	Intrarater reliability *(n = 534)*		Interrater reliability *(n = 137)*	
	Mean	SD	Mean	SD
Age (years)	19	0.6	19	0.4
Height (m)	1.81	0.07	1.81	0.06
Weight (kg)	77	14	78	10
BMI (kg/m^2^)	23.6	3.7	23.7	2.7

SD, standard deviation; BMI, body mass index.

### Data collection and test procedure

In the intrarater part of the study, the same rater administered the test on every participant on two occasions. In the interater part, two raters, independent of each other, administered the test on every participant once only. In both the intra- and interrater part, all participants conducted the two tests occasions on the same day with about two hours between tests. Raters and participants were blinded to the result from the first test when the second test was conducted.

#### The IsoKai lift test procedure

**Fig 2:**
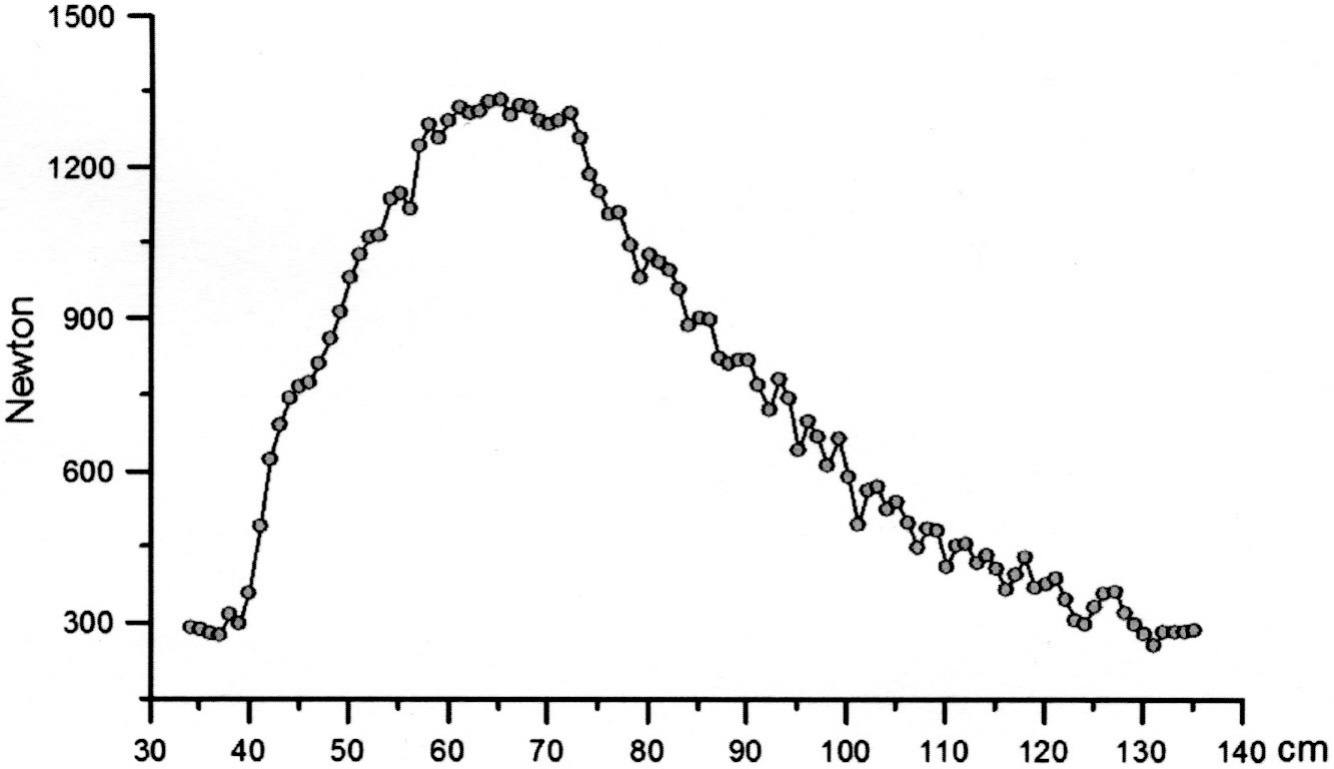
The force curve generated from an IsoKai isokinetic lift test.

IsoKai is a device used for measuring isokinetic muscular performance during a vertical lifting procedure. The device consists of a frame holding a vertical lifting bar that, via two wires, is connected to a hydraulic system regulating the speed of the lift at 0.30 m/sec. The IsoKai device allows a small horizontal movement (6–7 cm) of the vertical bar during the lift. The muscular force (N) is, for each cm of the lift, registered by a computer connected to a force plate on which the participant was instructed to stand with feet separated by shoulder width. The test was carried out from a start position where the back was straight while being forwardly inclined and knees bent. Thereafter, a two-handed lift of a vertical bar from 30 cm above the force plate was carried out until in upright standing with the bar at shoulder level (Fig 1). The participants were instructed to use maximal effort during the lift. No shoes were allowed during test. Before the test, participants performed a 10 minutes warm-up session using a cycle ergometer. Length was registered by an electronic measure instrument connected to the IsoKai device, and weight was registered by the force plate. This was done in order to adapt the IsoKai devise calculations to each participant. The rater then gave oral instructions on how to execute the lift, and, in addition, demonstrated the lift to secure a safe test. One sub-maximal practice test lift was allowed for the participant to get used to the testing procedure. Each test occasion consisted of two to four maximal lifts. For each lift, a graph in which the force was plotted against each cm of the lift was generated (Fig 2).

The IsoKai mean force (IsoKai_MF_) and peak force (IsoKai_PF_) measures generated at each lift were registered. The IsoKai_MF_ represents the mean of all the registered forces (plots in the force curve) during one lift, while the IsoKai_PF_ is the maximum force produced during the lift (the top of the force curve). Only the highest IsoKai_MF_ and IsoKai_PF_ measures produced during the test occasion were used as estimates in the analyses. Prior to the test occasion, the IsoKai device was calibrated by experienced personnel from the Swedish Defence Recruitment Agency.

### Statistical analyses

Descriptive data were presented with means and standard deviations (SD). The level of significance was set to 95%. Statistical analyses were performed using IBM SPSS Statistics version 23 (IBM Corporation, USA). See the S1 Appendix for the ANOVA results, and some example ICC calculations.

#### Reliability

The intraclass correlation coefficient (ICC) was calculated together with its corresponding 95% confidence interval (95% CI) [17–19]. The ICC_1,1_ equation was used for the intrarater reliability calculations, and the ICC_2,1_ equation for the interrater reliability calculations [17–19].

$$ICC_{1.1}=\;\left(BMS-WMS\right)/\left(BMS+WMS\right)$$

$$ICC_{2.1}=\left(BMS-EMS\right)/(BMS+\left(k-1\right)EMS+\;\frac{k}{n}\left(JMS-EMS\right))$$

The equation variables were estimated using repeated measures analysis of variance (ANOVA). BMS represents the between-people variability of the measurement, WMS the within-people variability, JMS the between-rater variability, EMS the residual mean square (error) variability, *k* the number of raters, and *n* the number of participants (S1 Appendix) [17–19]. An ICC coefficient of 1.0 indicates perfect reliability of a measurement while zero indicates a totally unreliable measurement [6]. Evaluation of the ICC coefficients was interpreted using the classification suggested by Cicchetti, with reliability coefficients categorised as: 0.75 to 1.0 = excellent, 0.60 to 0.74 = good, 0.40 to 0.59 = fair and less than 0.40 = poor [20].

#### Agreement

Agreement was assessed using the standard error of measurement (SEM) and the smallest real difference (SRD), along with their 95% CI [21–23]. The SRD shows the limit for the smallest difference (N) that indicates a real change for an individual. Further, the SEM% and SRD% were calculated to be able to evaluate the measurement error and individual changes independently from the units of measurement (N) [23]. SEM% and SRD% are recommended to be used for comparison with other studies, especially to account for the fact that different units of measurement are used [23].Formulas used to assess agreement:

$$SEM=\;\sqrt{WMS}$$

SRD=√2x1.96xSEM

$SRD\;95\%\;CI=\bar{d}\pm SRD$, where $\bar{d}$ represent the overall mean difference between two tests.

SEM%=(SEM/Grandmean)x100

SRD%=(SRD/Grandmean)x100

Grand mean = (the sample mean force of the IsoKai_MF_ or IsoKai_PF_ from test occasion 1 + the sample mean force of the IsoKai_MF_ or IsoKai_PF_ from test occasion 2) / 2.

#### Bland and Altman methods

Bland and Altman methods were used to assess the dispersion of data, measurement error and possible systematic bias of the test [7, 24]. These methods were based on the analysis of differences between measurements from the repeated test occasions. Using Bland and Altman plots, the mean values from two tests were plotted against the difference between the tests for each participant. The Bland and Altman plots visualised the distribution of the individual test differences around the overall mean difference between tests ($\bar{d}$) together with the limits of agreement (LOA).

The LOA which represented the precision of the measurement was calculated by the formula: $\bar{d}\pm 2\;standard\;deviations\;\left(SD\right)$ [24]. Further, as an overall mean difference between the tests occasions ($\bar{d}$) significantly different from zero indicates a systematic bias in the test, a possible bias was formally assessed by estimating $\bar{d}$ together with the 95% CI [18, 24, 25]. The 95% CI of $\bar{d}$ were calculated using the formula; $\bar{\;d}\pm \;t_{n-1}$, where *t*_*n-1*_ represents the probability point of the t distribution on n-1 degrees of freedom, and SE the standard error of $\bar{d}$[18].

## Results

Table 2 presents the sample mean force and the “Grand mean” for the IsoKai_MF_ and IsoKai_PF_ measures in the intra- and interrater samples respectively.

**Table 2:** The sample mean force for the IsoKai_MF_ and IsoKai_PF_ at the different test occasions and the “Grand mean” in the intra- and interrater samples.

	Intrarater reliability (*n = 534*)			Interrater reliability (*n = 137*)		
	Test 1	Test 2		Rater 1	Rater 2	
	Mean (SD)	Mean (SD)	G-mean (SD)	Mean (SD)	Mean (SD)	G-mean (SD)
IsoKai_MF_ (N)	711 (114)	717 (111)	714 (111)	757 (103)	752 (104)	754 (102)
IsoKai_PF_ (N)	1246 (216)	1260 (211)	1253 (206)	1368 (194)	1356 (192)	1362 (186)

IsoKai_MF,_ IsoKai mean force; IsoKai_PF,_ IsoKai peak force; N, Newton; G-mean = Grand mean = (sample mean force of the IsoKai_MF_/IsoKai_PF_ from test 1/rater 1 + sample mean force of the IsoKai_MF_/IsoKai_PF_ from test 2/rater 2) / 2.

### Reliability

All results showed that the IsoKai isokinetic lift test had excellent reliability, with ICC coefficients ranging from 0.858 to 0.942 (Table 3).

**Table 3:** Intraclass correlation estimates (ICC) for the IsoKai_MF_ and IsoKai_PF_ measures of the IsoKai isokinetic lift test for the intra- and interrater reliability analyses.

	**Intrarater reliability** (*n = 534*)		**Interrater reliability** (*n = 137*)	
	ICC_1.1_	95% CI	ICC_2.1_	95% CI
IsoKai_MF_	0.935	0.923 to 0.946	0.942	0.920 to 0.959
IsoKai_PF_	0.865	0.842 to 0.886	0.858	0.806 to 0.896

IsoKai_MF,_ IsoKai mean force; IsoKai_PF,_ IsoKai peak force; ICC_1.1_ and ICC_2.1_, intraclass correlation coefficient; 95% CI, 95% confidence interval.

### Agreement

The agreement estimates based on the ANOVA analyses (SEM, SEM%, SRD and SRD%) indicated a higher degree of agreement for the IsoKai_MF_ than IsoKai_PF_, and a higher degree of agreement for the interrater sample compared to the intrarater sample (Table 4).

**Table 4:** Agreement estimates for the IsoKai_MF_ and IsoKai_PF_ of the IsoKai isokinetic lift test for the intra- and interrater agreement part of the study.

	**Intrarater agreement** (*n = 534*)					**Interrater agreement** (*n = 137*)				
	G-mean N	SEM N	SEM% %	SRD (95% CI) N	SRD% %	G-mean N	SEM N	SEM% %	SRD (95% CI) N	SRD% %
IsoKai_MF_	714	29	4.0	79 (73 to 86)	11.1	754	25	3.3	69 (64 to 74)	9.2
IsoKai_PF_	1253	78	6.2	217 (203 to 231)	17.3	1362	73	5.4	202 (191 to 214)	14.8

IsoKai_MF,_ IsoKai mean force; IsoKai_PF,_ IsoKai peak force; N, Newton; G-mean = Grand mean = (sample mean force of the IsoKai_MF_/IsoKai_PF_ from test 1/rater 1 + sample mean force of the IsoKai_MF_/IsoKai_PF_ from test 2/rater 2) / 2; SEM, standard error of measurement; SEM%, (SEM/Grand mean) x 100; SRD, smallest real difference; 95% CI, 95% confidence interval; SRD%, (SRD/Grand mean) x 100.

### Bland and Altman methods

**Fig 3:**
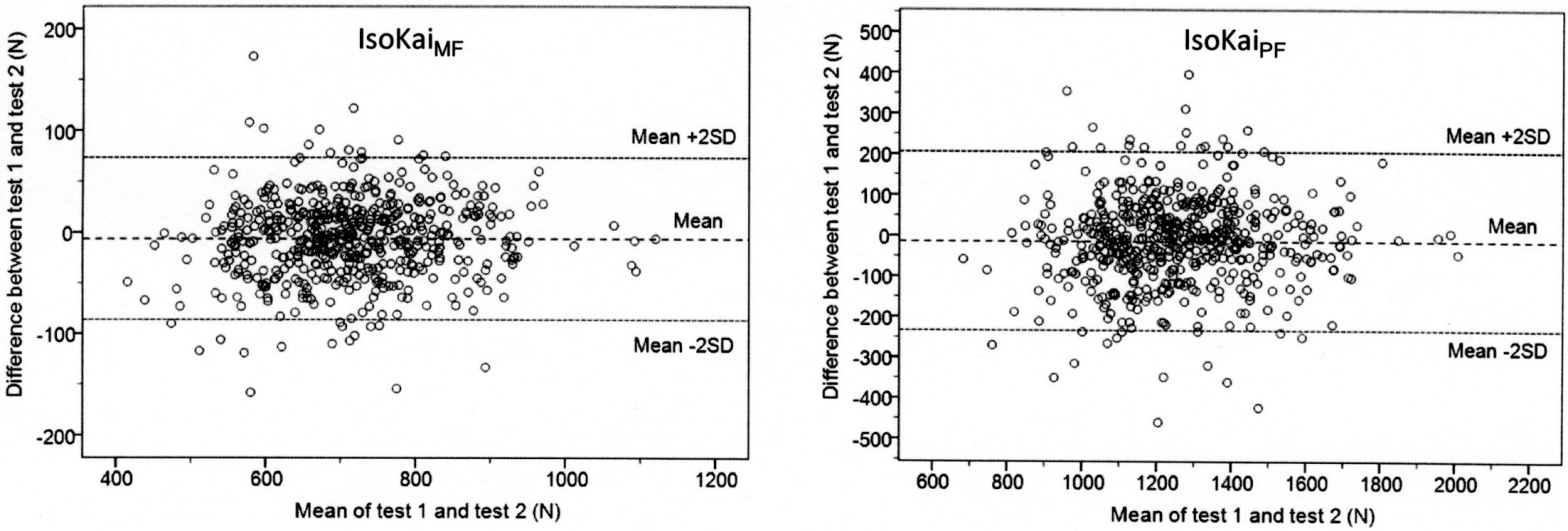
The mean of test 1 force and test 2 force plotted against the difference between the test 1 force and test 2 force in the intrarater sample. IsoKai_MF,_ IsoKai mean force (N); IsoKai_PF,_ IsoKai peak force (N); LOA, limits of agreement.

**Fig 4:**
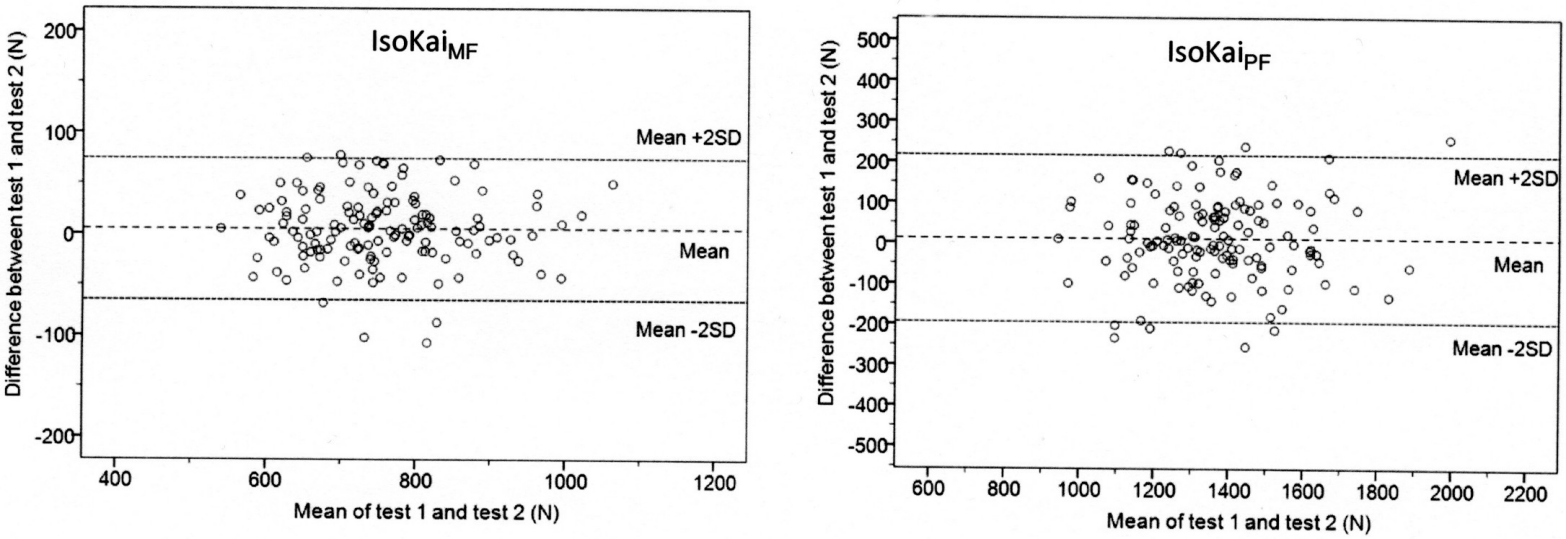
The mean of test 1 force (rater 1) and test 2 force (rater 2) plotted against the difference between test 1 force (rater 1) and test 2 force (rater 2) in the interrater sample. IsoKai_MF,_ IsoKai mean force (N); IsoKai_PF,_ IsoKai peak force (N); LOA, limits of agreement.

Bland and Altman results for the intra- and interrater samples are presented in Table 5 and Figs 3 and 4. The overall mean difference between tests ($\bar{d}$) in the intrarater sample was significantly different from zero, with—6 N (95% CI; -10 to -3) for the IsoKai_MF_ and—14 N (95% CI; -23 to -4) for the IsoKai_PF_.

**Table 5:** Bland and Altman measure for the intra- and interrater sample IsoKai_MF_ and IsoKai_PF_.

	**Intrarater sample** (*n = 534*)				**Interrater sample** (n = 137)			
	*$\bar{d}$*	95% CI	SE	SD	*$\bar{d}$*	95% CI	SE	SD
IsoKai_MF_ (N)	-6	-10 to -3	1.7	40	5	-1 to 11	3.0	35
IsoKai_PF_ (N)	-14	-23 to -4	4.8	110	12	-6 to 29	8.8	103

IsoKai_MF,_ IsoKai mean force; IsoKai_PF,_ IsoKai peak force; N, Newton; $\bar{d}$, overall mean difference between test/rater; 95% CI, 95% confidence interval; SE, standard error; SD, standard deviation.

## Discussion

This study revealed that the IsoKai isokinetic lift test is a highly reliable test for evaluating maximal dynamic muscular strength related to lifting. To establish if a measurement gives reliable information, assessing both measures of reliability and agreement is recommended [6, 7, 22]. Our analyses demonstrated that the IsoKai isokinetic lift test had excellent reliability for measuring maximal strength irrespective of whether the tests were performed by the same rater (intrarater reliability) or by two different raters (interrater reliability). Overall, the ICC coefficients showed that the test is an excellent test to use in order to rank male conscripts with regard to maximal muscular strength needed for lifting and similar tasks. Further, the IsoKai test was found to be an excellent test to distinguish between individuals assessing maximal strength when lifting from “floor” level to hip level (IsoKai_PF_; ICC_1.1_ = 0.865, ICC_2.1 =_ 0.858), as well as from “floor” level to shoulder level (IsoKai_MF_; ICC_1.1_ = 0.935, ICC_2.1 =_ 0.942). Even if only the IsoKai_MF_ is tested for content validity, we believe that the IsoKai_PF_ evaluates mainly low body and back strength, while the IsoKai_MF_ is a measure of whole body strength [14]. All the lower limits of the ICC confidence intervals exceeded 0.8 which further support the excellent reliability of the IsoKai isokinetic lift test.

There are no general recommendations set for how to evaluate agreement estimates as the evaluation depends on the context in which the test is used [7]. Therefore, the results in the present study could be evaluated in the light of finding potential changes in maximal muscular strength due to training or de-training [26]. The SEM and SEM% represent the error of the measurement. The SEM in the two samples were 29 and 25 N for the IsoKai_MF_, and 78 and 73 for the IsoKai_PF_. If taking the SEM of 29 N IsoKai_MF_ in the interrater sample as an example, this corresponds to a measurement error of only 3 kg (29N*0.102). We believe this supports the IsoKai test to be a rather precise measurement of changes in dynamic muscular strength. The precision of measuring individual changes represented by the SRD and SRD% was less pronounced, with SRDs between 69 to 217 N (Table 4), or 7 to 22 kg.

The Bland and Altman analyses showed no major signs of systematic errors in either the intrarater sample or the interrater sample. The plotted differences between two tests were evenly distributed about the overall mean difference ($\bar{d}$) in all analyses. In the intrarrater sample a statistically significant difference from zero was found specifically for the mean difference between tests, with values of -6 N and -14 N for both the IsoKai_MF_ and IsoKai_PF_, respectively (Table 5). This corresponded to about 1% of the “Grand mean” force values; a minor systematic error which could be negligible in this case. Concerning the interrater sample, about the same differences from zero as in the intrarater sample were found for the overall mean difference ($\bar{d}$), however, these were not statistically significant.

### Comparison with other studies

Isokinetic strength measures have been criticised as they mostly engage only one or two joints and their associated muscle groups which bear little relationship to the multi-joint and multi-muscle actions that take place during movements in real practice [4]. As such, the IsoKai lift isokinetic lift test is unique among isokinetic tests as it engages almost all muscle groups and joints in the body in a movement imitating a normal lifting procedure. As mentioned in the introduction, Larsson et al. (2009) assessed the reliability of the IsoKai test as it was performed in the SwAF before 2013 [16]. By using the mean value of two IsoKai_MF_ registrations as a measure of muscle strength capacity in a sample of Swedish conscripts (n = 427), they found an ICC_3.1_ of 0.94, a SEM of 30 and a SEM% of 4.3, findings very similar to ours. The IsoKai_PF_ was not evaluated. In addition to this study, we found two reliability evaluations of multi-joint isokinetic tests similar to the IsoKai isokinetic lift test [27, 28]. Bridgeman et al. found an isokinetic multi-joint squat devise to have good test-retest reliability, which was evaluated within 3 test sessions over a 3-week period in a sample of 10 strength trained male athletes. The concentric peak force (N) outcome revealed ICCs ranging from 0.87 to 0.98, and coefficients of variation (CV) from 7.6 to 15.4. The CV was calculated by dividing the method error (ME) by the overall mean difference of two tests ($\bar{d}$) and then multiply by 100 [23]. Lexell and Downham states that “if the sample size is sufficiently large and the mean difference small, both highly likely conditions, ME and SEM take similar values”, indicating that CV and SEM% could be comparable between studies [23]. In 1997, Wilson et al. examined another isokinetic squat device by measuring concentric peak force (N) in 29 athletic male subjects performing two tests with 3 minutes rest in-between, and found an ICC of 0.89 and a CV of 8.7 [27]. The IsoKai_PF_ measures in the current study estimated a muscular force comparable with the outcome in the above mentioned squat tests, and resulted in ICCs of the same magnitude (0.858 and 0.865, Table 3). The IsoKai_PK_ SEM% of 5.4 and 6.2 indicate better precision of the IsoKai lift test compared to the CVs from the squat tests evaluations; however, such a comparison should be done with caution since the small sample sizes might have influenced the results.

### Methodological considerations

Today many women apply for enrolment in physically demanding occupations such as in the police force and military service. As such, the lack of women in our samples could be regarded as a limitation. However, unpublished data from the SwAF indicates excellent ICC values for women (n = 18) regarding the IsoKai_MF_ (ICC_1.1_ and ICC_2.1_ of 0.811) and IsoKai_PF_ (ICC_1.1_ and ICC_2.1_ of 0.767). Given these preliminary results, we find no reason to believe that the IsoKai isokinetic lift test would not be a reliable test among women. The data in the present study might be regarded as old since it dates back to 2001 and 2002. Still, as the IsoKai device used at time of data collection was exactly the same type as used today, we believe that the results are still applicable. There are several suggested ICC equations recommended in the literature depending on the context of the test procedures, but no standardised system on how to choose the most appropriate ICC exists [7, 17, 18]. We decided to use the ICC_1,1_ equation (intrarater reliability) and the ICC_2,1_ equation (interrater reliability) in the analyses, as they corresponded well to recommendations when compared to the test proceedings in which the IsoKai lift test was performed, and how it is used in the Swedish Defence Recruitment Agency [17–19, 29]. A number of arbitrary recommendations exist regarding the interpretation of satisfactory levels of reliability of a test when using the ICC. Resultantly, we chose the recommendations by Ciccetti [20]. Portney and Watkins suggest that an ICC above 0.75 indicate good reliability, while Currier et al suggest coefficients ranging from 0.80 to 0.90 indicate good reliability and above 0.90 indicate high reliability [6, 30]. No matter which of these recommendations used, the IsoKai isokinetic lift test would be regarded as a test with good-to-high levels of reliability even if the lower limit of the CI is considered, as is recommended [7, 29]. The overall lower ICCs and larger agreement measures for the IsoKai_PF_ compared to the IsoKai_MF_ might be explained by the fact that the IsoKai_PF_ is registered momentarily while the IsoKai_MF_ is averaged during the completed lift. Therefore, the measurement error inherent to the device itself, or differences in the momentarily force produced by the participant, might result in a larger WMS and EMS estimates for the IsoKai_PF_ than the IsoKai_MF_, causing these differences [22]. This might also affect the relationship between the BMS and the WMS/EMS, as reflected in the F statistics of the ANOVA (S1 Appendix). A high F statistics indicates a large discrepancy between the BMS and WMS/EMS, as in the IsoKai_MF_, which results in high ICC measures, while a lower F statistics, as in the IsoKai_PK_, results in lower ICC measures [6, 22, 31]. We believe that our sample sizes are considered a strength as it widely exceeds recommendations for reliability studies. Bonett et al. suggests a sample size of 108 subjects in order to detect an ICC of 0.70 or higher with a power of 0.80 and an alfa level set to 0.05 [32]. With the same power and alfa level, Walter et al. estimated a sample of 117 subjects is required to find an expected reliability of 0.80 [33]. Bonnet et al. further emphasise that it is unnecessary to include more subjects than needed for the desired power, as it increases the costs of research, and do not necessarily improve the results [32]. We fully agree, but as the data in our study already were collected as a part of the SwAF admission procedure, we found no reason not to use the entire sample. Another strength is that our data were collected in a way that Carter et al. call a “partially standardised approach” which has the purpose of describing reliability with the levels of standardisation that could be achieved in real settings [31]. We believe this to further supports the findings of the IsoKai lift test as a reliable measurement when implemented into real practice. Finally, we have fulfilled all the requirements in the GRRAS checklist for reporting of studies of reliability and agreement [7].

### Implications of the results

Military personnel are often exposed to high physical demands in their work tasks, especially during military missions [34–36]. Common tasks during military service include marching, digging, carrying and lifting, with lifting to be the most frequent work task [11, 34, 37]. In 2015, Larsson et al. found that the IsoKai isokinetic lift test had excellent content validity with respect to digging, carrying and lifting tasks [14]. Hence, to use it as an admission test in military settings and other physically demanding occupations with similar work tasks would be of great benefit. Further, as we evaluated the IsoKai isokinetic test using a representative population in comparison with the target population, the results support the use of the test in military settings as well as other similar settings e.g. the police force. The IsoKai lift test is fast, easy to assess and relatively safe as the resistance in the isokinetic test adapts to muscle force [2, 5]. This makes it an excellent test in selection procedures targeting large groups especially where time constraints exist. The test also has some drawbacks that have to be mentioned. The device is difficult to transport, needs experienced personnel for calibration, and is quite costly. It is important to notice that the reliability estimates are dependent of the variability of the outcome in the population measured, which might limit the external validity [7, 22]. The mean age in our samples was 19 years, which could be argued to restrict our findings to a young population. However, even if our samples are homogenous regarding age, the between subject variability (BMS) of the outcome showed large heterogeneity in all our analyses (S1 Appendix). Therefore, our results could probably be valid even for other age categories as it is not the actual age that matters but the variance of the measures when evaluating reliability [6]. The IsoKai isokinetic lift test could also be used to assess clinical important changes in muscular strength in individuals following an intervention, for example in sport or sports medicine. Measures of agreement, such as the SEM and the SRD, are not dependent on the variability between subjects, since only the measurement error is of importance [22]. Therefore, measures of agreement could to a greater extent be used in various populations other than reliability measures.

### Recommendations for future research

The constant speed and varying resistant in an isokinetic strength test have advantages, as discussed earlier, but could also be a disadvantage in relation to muscle performance in real practice where speed typically varies and load remains constant. This could challenge the validity of the IsoKai isokinetic lift test in comparison to isoinertial lifting capacity. One possible solution to this could be to investigate the criterion or concurrent validity of the IsoKai lift test in relation to maximal strength measured with an isoinertial lift test, for example a one repetition maximum lift test. Moreover, we believe that examining the reliability of the IsoKai isokinetic lift test in other populations would be beneficial in order to increase the external validity of the test.

### Conclusion

The IsoKai isokinetic lift test was found to be a highly reliable measurement of maximal dynamic muscular strength. The test could be used to monitor dynamic muscular strength for the purpose of distinguishing between individuals. As such, the IsoKai isokinetic lift test could be recommended for use as a test for selection based on capacity levels regarding maximal dynamic muscular strength in military settings as well as other physically demanding occupations. In addition, the test is useful for evaluating changes in maximal muscle strength in individuals following interventions.

## Supporting information

S1 Appendix (DOCX)

S1 Data IsoKai intrarater reliability. (XLSX)

S2 Data IsoKai interrater reliability. (XLSX)
