# Supplementary material for: Reliability and agreement of the IsoKai isokinetic lift test – A test used for admission to the Swedish Armed Forces
Source: PLoS One. 2018 Dec 19;13(12):e0209419. doi: 10.1371/journal.pone.0209419 (PMC6300333; doi:10.1371/journal.pone.0209419)
Supplement: S1 Appendix — (DOCX) [file pone.0209419.s001.docx]

## Appendix

Statistical analysis was performed using IBM SPSS Statistics version 23 (IBM Corporation, USA).

### Reliability

Results from the SPSS repeated measurements ANOVA used to calculate the ICC_1.1_ for the intrarater reliability and the ICC_2.1_ for the interrater reliability. Also presented are examples of the ICC calculations: ICC_1,1_ = (BMS – WMS) / (BMS + WMS)

ICC_2,1_ = (BMS – EMS) / (BMS + (*k-*1)EMS + $\frac{k}{n}$(JMS – EMS))

BMS represents the between-subject (between people) variability of the measurement, WMS the within-subject (within people) variability, JMS the between rater variability, EMS the residual mean square (error) variability, *k* the number of items/raters an *n* the number of subjects.

| **Intrarater reliability**  **ANOVA** | | | | | | |
| --- | --- | --- | --- | --- | --- | --- |
| **IsoKai mean force values** | | Sum of Squares | df | Mean Square | F | Sig |
| Between People (BMS) | | 13021177,461 | 533 | 24429,976 |  |  |
| Within People | Between Items | 10697,004 | 1 | 10697,004 | 13,426 | ,000 |
|  | Residual | 424646,996 | 533 | 796,711 |  |  |
|  | Total (WMS) | 435344,000 | 534 | 815,251 |  |  |
| Total | | 13456521,461 | 1067 | 12611,548 |  |  |

| **ANOVA** | | | | | | |
| --- | --- | --- | --- | --- | --- | --- |
| **IsoKai peak force values** | | Sum of Squares | df | Mean Square | F | Sig |
| Between People (BMS) | | 45212280,996 | 533 | 84826,043 |  |  |
| Within People | Between Items | 50748,169 | 1 | 50748,169 | 8,397 | ,004 |
|  | Residual | 3221400,831 | 533 | 6043,904 |  |  |
|  | Total (WMS) | 3272149,000 | 534 | 6127,620 |  |  |
| Total | | 48484429,996 | 1067 | 45439,953 |  |  |

IsoKai peak force (IsoKai_Peak_):
ICC_1,1_ = BMS – WMS / BMS + WMS = 84826.043 – 6127.620 / 84826.043 + 6127.620 = 0.865
The ICC_1,1_ and the 95% CI were estimated using the SPSS reliability analyses with a one-way random effect model.

| **Interrater reliability**  **ANOVA** | | | | | | |
| --- | --- | --- | --- | --- | --- | --- |
| **IsoKai mean force values** | | Sum of Squares | df | Mean Square | F | Sig |
| Between People (BMS) | | 2844606,985 | 136 | 20916,228 |  |  |
| Within People | Between Items (JMS) | 1752,734 | 1 | 1752,734 | 2,860 | ,093 |
|  | Residual (EMS) | 83358,766 | 136 | 612,932 |  |  |
|  | Total | 85111,500 | 137 | 621,252 |  |  |
| Total | | 2929718,485 | 273 | 10731,570 |  |  |

| **ANOVA** | | | | | | |
| --- | --- | --- | --- | --- | --- | --- |
| **IsoKai peak force values** | | Sum of Squares | df | Mean Square | F | Sig |
| Between People (BMS) | | 9434913,839 | 136 | 69374,366 |  |  |
| Within People | Between Items (JMS) | 9168,704 | 1 | 9168,704 | 1,732 | ,190 |
|  | Residual (EMS) | 719853,796 | 136 | 5293,043 |  |  |
|  | Total | 729022,500 | 137 | 5321,332 |  |  |
| Total | | 10163936,339 | 273 | 37230,536 |  |  |

IsoKai peak force (IsoKai_Peak_):
ICC_2,1_ = BMS – EMS / BMS + (*k-*1)EMS + $\frac{k}{n}$(JMS – EMS) = 69374.366 – 5293.043 / 69374.366 + 5293.043 + $\frac{2}{137} ($9168.704 – 5293.043) = 0.858

The ICC_2,1_ and the 95% CI were estimated using the SPSS reliability analysis with a two-way random effect model and the absolute agreement definition.
